# Peristomal granulation tissue mimicking peristomal pyoderma gangrenosum in the context of urine-diverting ostomies

**DOI:** 10.1016/j.jdcr.2022.08.040

**Published:** 2022-08-27

**Authors:** Brett D. McLarney, Simo Huang, Sylvia Hsu

**Affiliations:** Department of Dermatology, Temple University Lewis Katz School of Medicine, Philadelphia, Pennsylvania

**Keywords:** peristomal pyoderma gangrenosum, peristomal ulcers, IBD, inflammatory bowel disease, PG, pyoderma gangrenosum, PPG, peristomal pyoderma gangrenosum

## Introduction

An estimated 1 million North Americans live with ostomies, with up to 80% developing stoma-related skin morbidities.^1^ Peristomal pyoderma gangrenosum (PPG), a subset of the neutrophilic dermatosis pyoderma gangrenosum (PG), is commonly included in the differential diagnosis for peristomal ulcers. Treatments for PPG, such as systemic immunosuppressive therapy, carry morbidity and can delay wound healing when PPG is not the correct diagnosis. With high numbers of patients experiencing peristomal skin lesions, erroneous diagnoses of PPG given to non-PPG peristomal lesions could lead to significant morbidity.^2^ While a Delphi consensus has standardized the diagnosis of PG (Table I), the literature continues to demonstrate the low performance of the Delphi criteria.^3^^,^^4^

**Table I:** Delphi criteria for diagnosing PG∗†

Major criterion
Biopsy with neutrophilic infiltrate
Minor criteria
Exclusion of infection on histology
Pathergy
Personal history of IBD or inflammatory arthritis
Papule, pustule, or vesicle that rapidly ulcerates
Peripheral erythema, undermining border, and tenderness at the site of ulceration
Multiple ulcerations (at least 1 occurring on an anterior lower leg)
Cribriform or wrinkled paper scars at healed ulcer sites
Decrease in ulcer size within 1 month after immunosuppressive treatment

*IBD*, Inflammatory bowel disease.

∗ In the Delphi exercise, a threshold of 1 major criterion and 4 of 8 minor criteria maximized diagnostic discrimination.

† Table adapted from Maverakis et al.^3^

Additionally, several other factors may increase the misdiagnosis of peristomal ulcers as PPG:

1. The etiology of PPG is poorly understood.^5^
2. Pathergy and the inconvenience of manipulating ostomy appliances both discourage biopsy of suspected lesions; thus, a large portion of PPG diagnoses is made clinically.
3. PPG's response to treatment is variable across literature reports, so treatment failure provides little reason for clinicians to suspect misdiagnosis.^2^
4. Several clinical characteristics of PPG, such as time from ostomy placement to ulceration, vary considerably across literature reports, which provides enough ambiguity for confirmation bias to support a misdiagnosis.^5^, ^6^, ^7^
5. Atypical presentations of common peristomal skin pathology, such as contact dermatitis and hypergranulation, can be convincing mimics of PPG.^5^

Herein, we present a case of peristomal granulation tissue that was originally referred to our dermatology clinic with the presumptive diagnosis of PPG.

## Case

**Fig 1:**
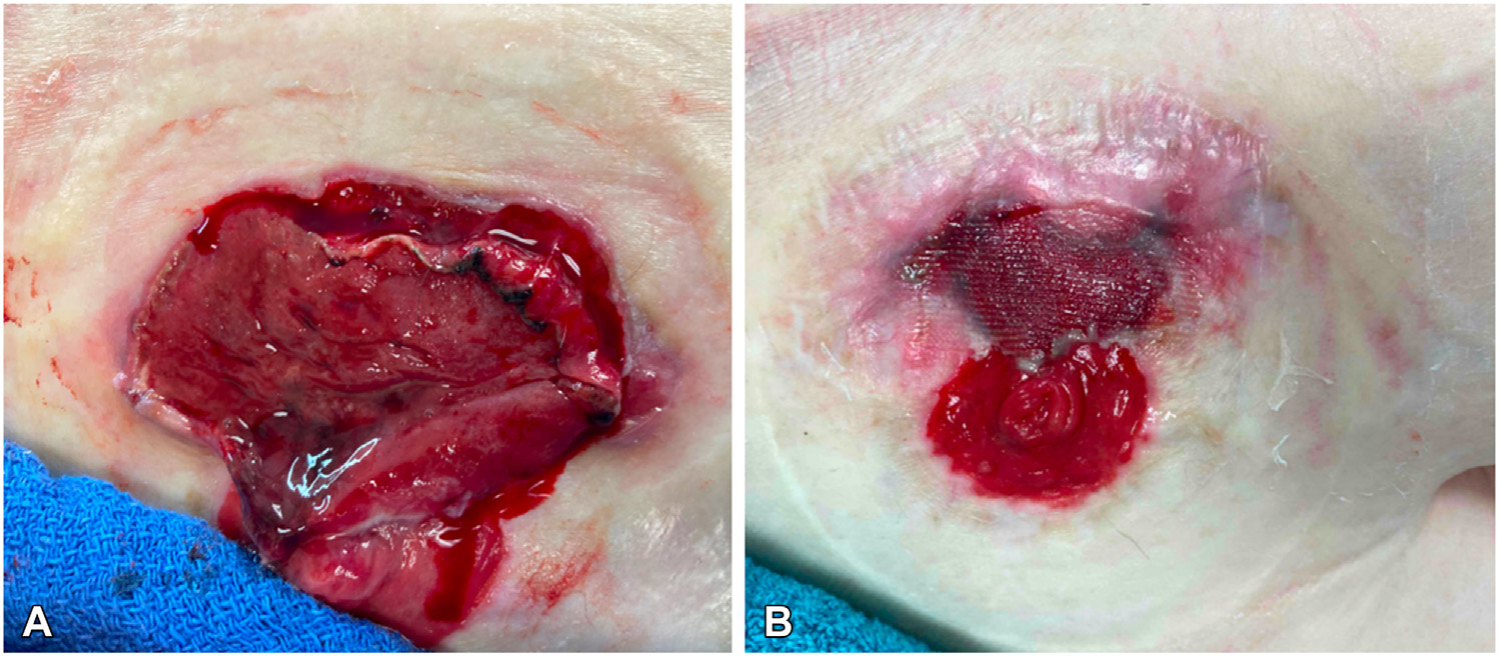
Patient’s peristomal ulcer (**A**) at the time of presentation and (**B**) 10 months after the initial presentation, following debridement, closure, and wound care managed by a plastic surgery team.

A 70-year-old man with a history of bladder malignancy presented with a slowly expanding peristomal ulcer 6 months after undergoing radical cystoprostatectomy and creation of an ileal loop urinary diversion. The patient reported that 1 month after ileostomy placement a "pimple" formed that opened and developed into a painful ulcer. The patient endorsed a poorly fitting ostomy apparatuses and frequent leakage of urine from the ostomy; he denied any history of inflammatory bowel disease (IBD) or autoimmune disease. The patient’s wound care regimen consisted of daily sodium chloride-impregnated dressing changes, daily topical collagenase ointment, and in-office cauterization of the wound with silver nitrate every 2 weeks. Wound cultures taken at another facility 2 months prior grew *Klebsiella pneumoniae*, *Enterococcus faecalis*, and *Staphylococcus aureus*, but the patient had not been treated with antibiotics. Visual inspection revealed an ileostomy site in the right upper quadrant and a 6 cm × 4 cm ulcer at the opening of the ostomy site (Fig 1, *A*). Punch biopsies of the medial and lateral borders of the wound were obtained, and both demonstrated prominent granulation tissue absent of neutrophilic infiltrate or evidence of infection. The patient was referred to plastic surgery who debrided and closed the peristomal ulcer. A subsequent wound care regimen consisting of silver sulfate foam bandages, sodium chlor-hypochlorous acid 0.033% solution, and daily collagenase ointment resulted in improvement of the ulcer (Fig 1, *B*).

## Discussion

Concern has been raised in the literature regarding PPG’s misdiagnosis and possible overdiagnosis.^2^ Given the unreliability and morbidity associated with PPG treatments, misdiagnosing peristomal lesions as PPG has deleterious consequences for the patient. Thus, it is important for dermatologists to recognize when a PPG mimic (Table II) is, in fact, not PPG. We highlight the criteria for PG met by this case alongside some red flags suggesting “not PPG.”

**Table II:** Peristomal skin pathology that can mimic PPG∗

Trauma
Infection or abscess
Ischemia
Hidradenitis suppurativa
Contact dermatitis from ostomy appliance or leakage
Drug-induced or exogenous tissue injury
Drug-induced lupus
Hydroxyurea induced
Injection drug abuse
Brown recluse spider bite
Factitious
Folliculitis
Hematoma
Cutaneous inflammatory bowel disease
Early enterocutaneous fistula formation
Vasculitis
Necrotizing fasciitis
Progressive bacterial synergistic gangrene
Autoimmune blistering disease
Malignancy
Other neutrophilic dermatoses
Sweet syndrome
Panniculitis
Acneiform lesions

*PPG*, Peristomal pyoderma gangrenosum.

∗ Table adapted from Afifi et al.^5^

Three Delphi consensus minor criteria for PG were met by our case: (1) the lesion was initially a papule that ulcerated, (2) histopathology excluded infection, and (3) peripheral erythema, tenderness, and an undermined border. The major criterion for PG, histopathology demonstrating neutrophilic infiltrate, was not present; however, PG in occluded and intertriginous areas can be stalled and develop granulation tissue, which may account for a modest proportion of PG cases that do not meet the consensus criteria. While our patient may be such a case of PG, several factors mitigate this suspicion. First, the patient had no history of either Crohn's disease or ulcerative colitis, yet the literature reports that PPG presents nearly exclusively in the context of IBD.^6^^,^^7^ The patient had a history of bladder malignancy; however, PPG’s association with malignancy is weaker. In fact, while over 75% of stomata are placed for colorectal carcinoma, malignancy with no history of IBD is noted as the underlying condition in very few (0% to 8%) of PPG cases.^6^, ^7^, ^8^ Secondly, our 70-year-old patient was older than the typical age of PPG presentation, which is 43-48 years.^5^, ^6^, ^7^ Lastly, the most common stoma associated with PPG is an ileostomy, which is quoted to represent 78% of the stomas associated with PPG.^5^ The literature speculates that ileostomies provide a particular enzyme- and/or cytokine-rich output, likely related to comorbid IBD, that favors PPG formation.^5^ However, while case 1 (Table III) had an ileostomy, it was a urine-diverting loop ileostomy; thus, the ostomy output would be primarily urine, rather than small intestine content. In our patient, the etiology of the ulcer was never definitively determined.

**Table III:** Reported cases and characteristics of PPG in urine-carrying ostomies

Case	Age, sex	Time to ulcer onset	Underlying disease	Stoma type	Biopsy	Effective treatment (time to resolution)
0∗	70, M	1 mo	Bladder cancer	Ileal loop urinary diversion	Granulation tissue	Surgical debridement and closure followed by wound care (10 mo)†
1^9^	83, M	20 y	Bladder cancer	Urostomy	Nonspecific inflammatory infiltrate	Triamcinolone acetonide, topical clobetasol (1.5 mo)
2^9^	73, M	11 mo	Bladder cancer	Urostomy	Nonspecific inflammatory infiltrate	1st occurrence: dapsone (11 mo) 2nd occurrence: mycophenolate mofetil (8 mo)
3^10^	45, F	5 wk	Neurogenic bladder	Urostomy	Granulation tissue	Tacrolimus 0.3% in carmellose sodium paste (1 mo)
4^10^	57, F	1 y	Bladder cancer	Urostomy	Granulation tissue	Minocycline twice daily while weaning the patient’s dose of daily prednisolone from 30 mg to 0 mg (1.5 mo)
5^10^	57 F	3 y	Neurogenic bladder	Urostomy	Not performed	2 months of no treatment followed by clobetasol propionate 0.05% for 2 weeks (2.5 mo)

*PPG*, Peristomal pyoderma gangrenosum.

∗ Denotes the patient in our case report.

† Extent of improvement shown in Fig 1, *B*. Wound care regimen: silver sulfate foam bandages, sodium chlor-hypochlorous acid 0.033% solution, and daily collagenase ointment.

Five cases of PPG associated with urostomies are reported in the literature (Table III).^9^^,^^10^ Three cases were associated with urostomies for bladder carcinoma, while 2 were associated with urostomies for neurogenic bladder. None reported a history of IBD. The average age of PPG diagnosis for these cases was 63 years old, which is closer to our 70-year-old patient's age than the average for PPG.^5^, ^6^, ^7^ Four cases were biopsied, 2 of which showed prominent granulation tissue without suggestive histological evidence for PPG; another 2 were reported as nonspecific inflammatory infiltrate.

## Conclusion

In our patient and the cases reviewed, age, medical history, stoma type, and histology differ from the classic characteristics of PPG enough to question whether PPG-appearing lesions around urostomy or urine-carrying stomata are truly PPG. These cases recapitulate the need for definitive diagnostic criteria for PPG. Before such diagnostic criteria become available, we recommend obtaining biopsies of suspected PPG, especially for ulcers around urostomy stomata. Ulcers not demonstrating neutrophilic infiltrate fail to meet the major criterion for PG, and management of these ulcers as PG should not proceed without a strong overriding clinical justification.

## Conflicts of interest

None disclosed.
